# Supplementary material for: MyD88 promotes myoblast fusion in a cell-autonomous manner
Source: Nat Commun. 2017 Nov 20;8:1624. doi: 10.1038/s41467-017-01866-w (PMC5696367; doi:10.1038/s41467-017-01866-w)
Supplement: Supplementary file 1 — Supplementary Information [file 41467_2017_1866_MOESM1_ESM.pdf]

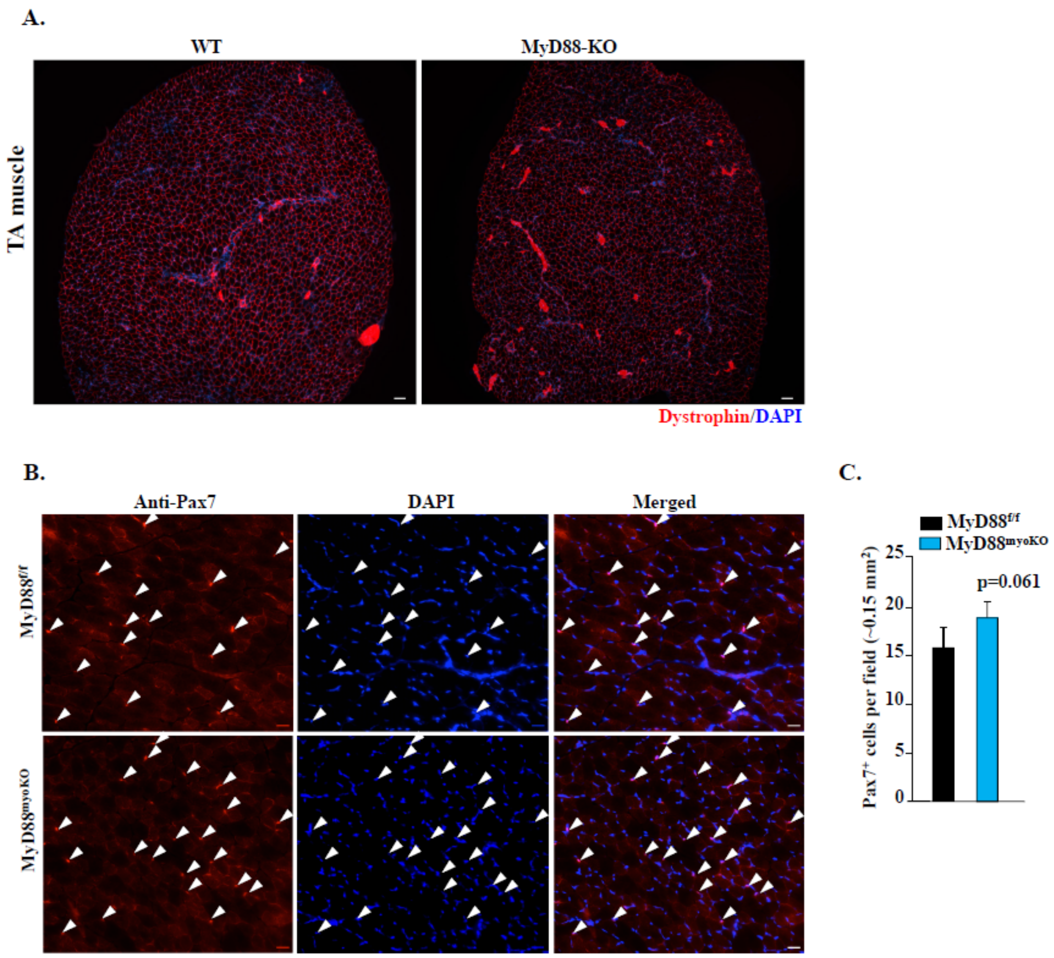

Supplementary Figure 1. MyD88 mediates postnatal muscle growth in mice. **(A)** TA muscle isolated from 2-week old WT and MyD88-KO mice were stained for dystrophin. Nuclei were identified by staining with DAPI. Representative images of muscle sections are presented here. Scale bar: 50 µm. **(B)** TA muscle sections from 2-week old $Myd88^{f/f}$ and $Myd88^{myoKO}$ mice were immunostained for Pax7 protein. Nuclei were counterstained by DAPI. Representative images are presented here. Arrows point to $Pax7^{+}$ cells. Scale bar: 20 µm. **(C)** Quantification of number of $Pax7^{+}$ cells per unit area (~0.15 mm^2) in TA muscle of 2-week old $Myd88^{f/f}$ and $MyD88^{myoKO}$ mice. Error bars represent s.d. N=4 in each group. No statistical difference was observed in number of $Pax7^{+}$ cells by unpaired t-test.

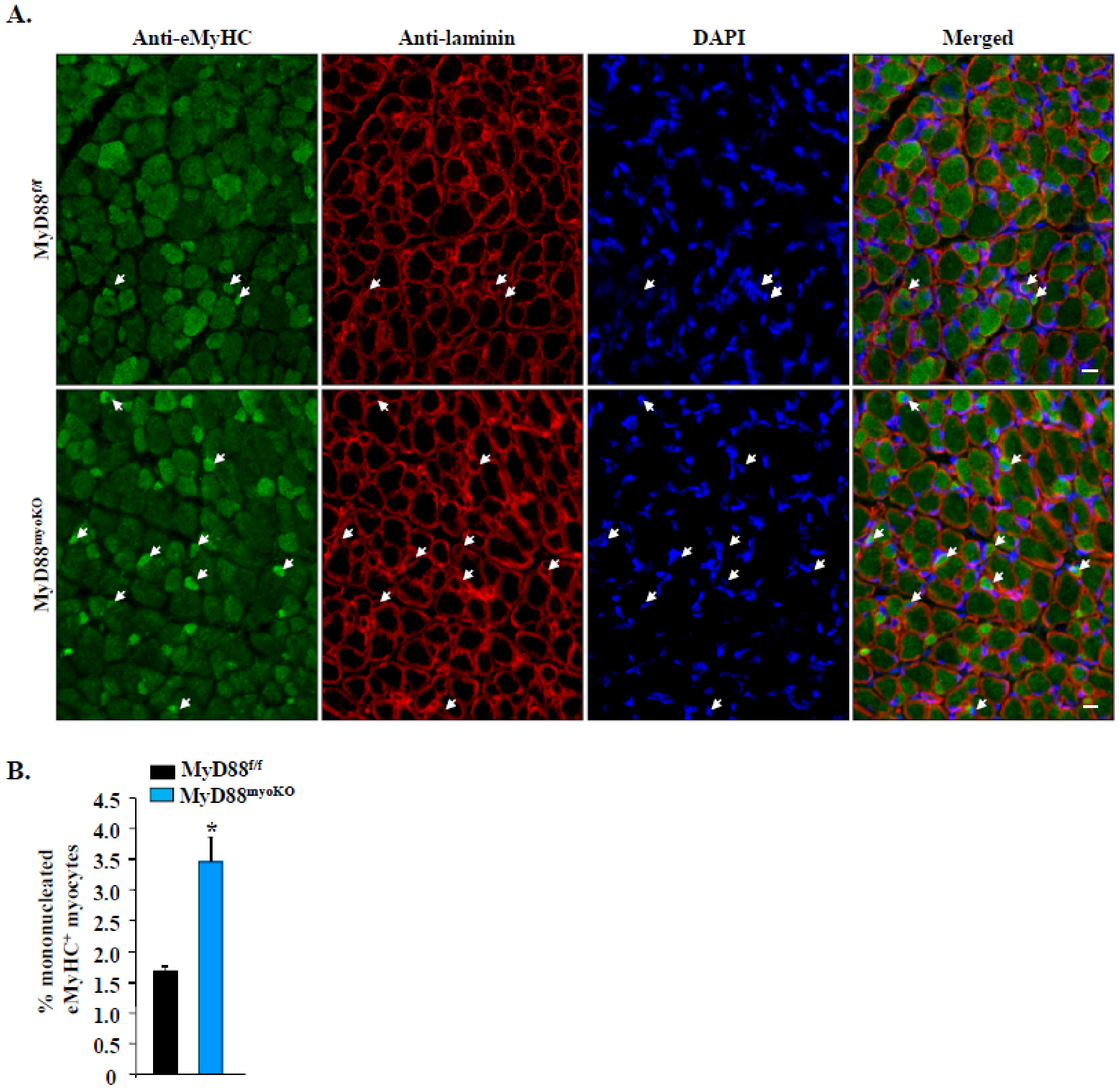

Supplementary Figure 2. Increased number of mononucleated myocytes in TA muscle of 5-day old MyD88^{myoKO} mice. (A) TA muscle isolated from 5-day old littermate MyD88$^{f/f}$ and MyD88^{myoKO} mice were immunostained for eMyHC and laminin proteins. Nuclei was counterstained with DAPI. Representative individual and merged images are presented here. Arrows point to mononucleated myofibers. Scale bar: 10µm. **(B)** Quantification of percentage of mononucleated eMyHC^{+} myofibers in TA muscle sections of 5-day old MyD88$^{f/f}$ and MyD88^{myoKO} mice. N= 4 in each group. *$p < 0.05$ from MyD88$^{f/f}$ mice by unpaired t-test.

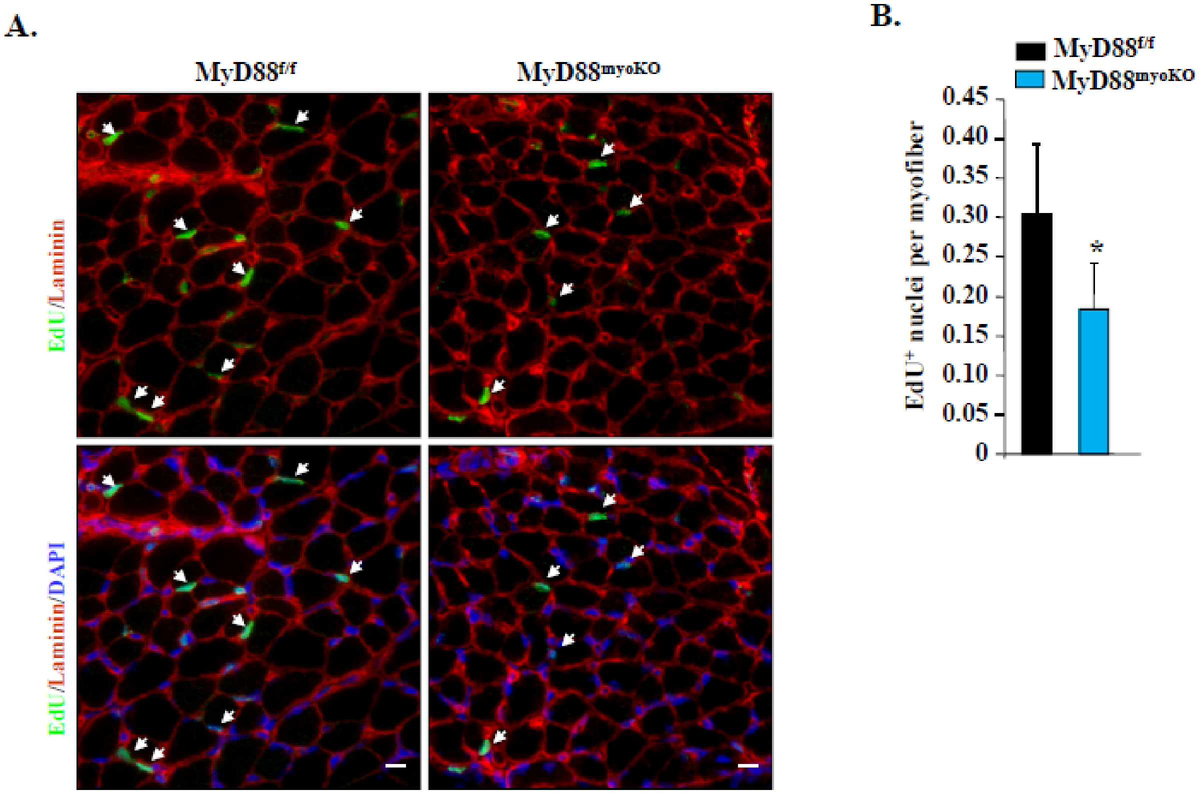

Supplementary Figure 3. Ablation of MyD88 reduces myoblast fusion during postnatal growth period. (A) Littermate MyD88$^{f/f}$ and MyD88^{myoKO} mice were given an intraperitoneal injection of EdU at P5 and P8. After 9 days of the first EdU injection, TA muscle was collected and muscle sections prepared were stained to detect EdU, laminin, and nuclei. Representative photomicrographs after EdU, laminin, and DAPI staining are presented here. Arrows point to EdU^{+} nuclei within myofibers. Scale bar: 10 µm. **(B)** Quantification of the percentage of EdU^{+} nuclei per myofiber in TA muscle sections. N=4-6 in each group. *$p < 0.05$ from MyD88$^{f/f}$ mice by unpaired t-test.

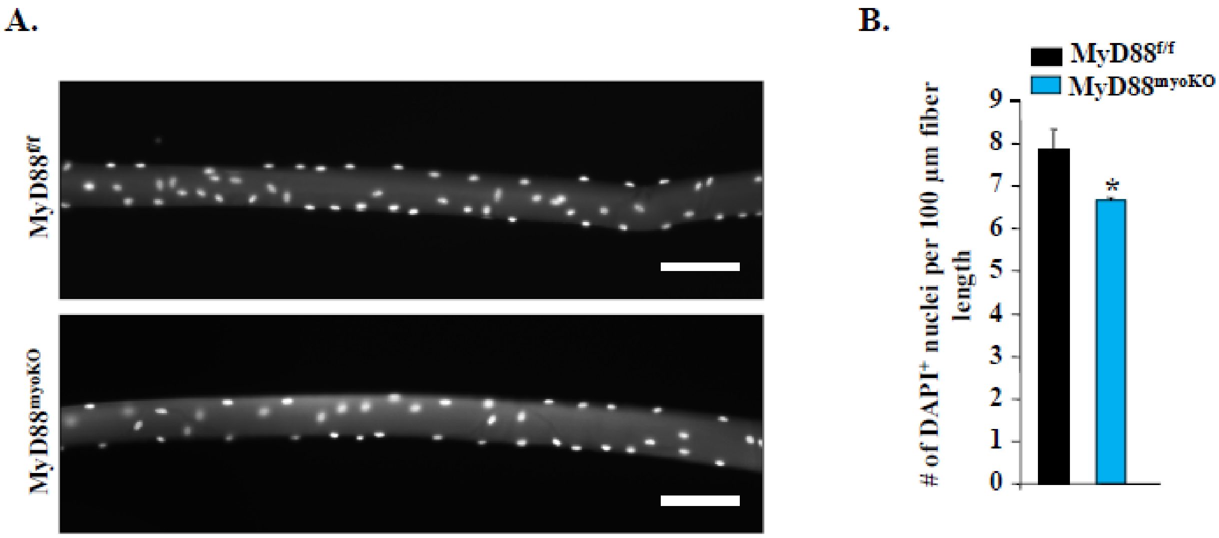

Supplementary Figure 4. Loss of MyD88 reduces number of myonuclei per myofiber in 3-month old mice. Single myofibers were prepared from EDL muscle of 3-month old MyD88$^{f/f}$ and MyD88^{myoKO} mice. Immediately after isolation, the myofibers were fixed and stained with DAPI to identify nuclei. **(A)** Representative DAPI stained myofibers, and **(B)** quantification of number of nuclei per unit (100 μm) myofiber length are presented here. Scale bar: 100 μm. N=4 in each group. Error bars represent s.d. *$p < 0.05$ from MyD88$^{f/f}$ mice by unpaired t-test.

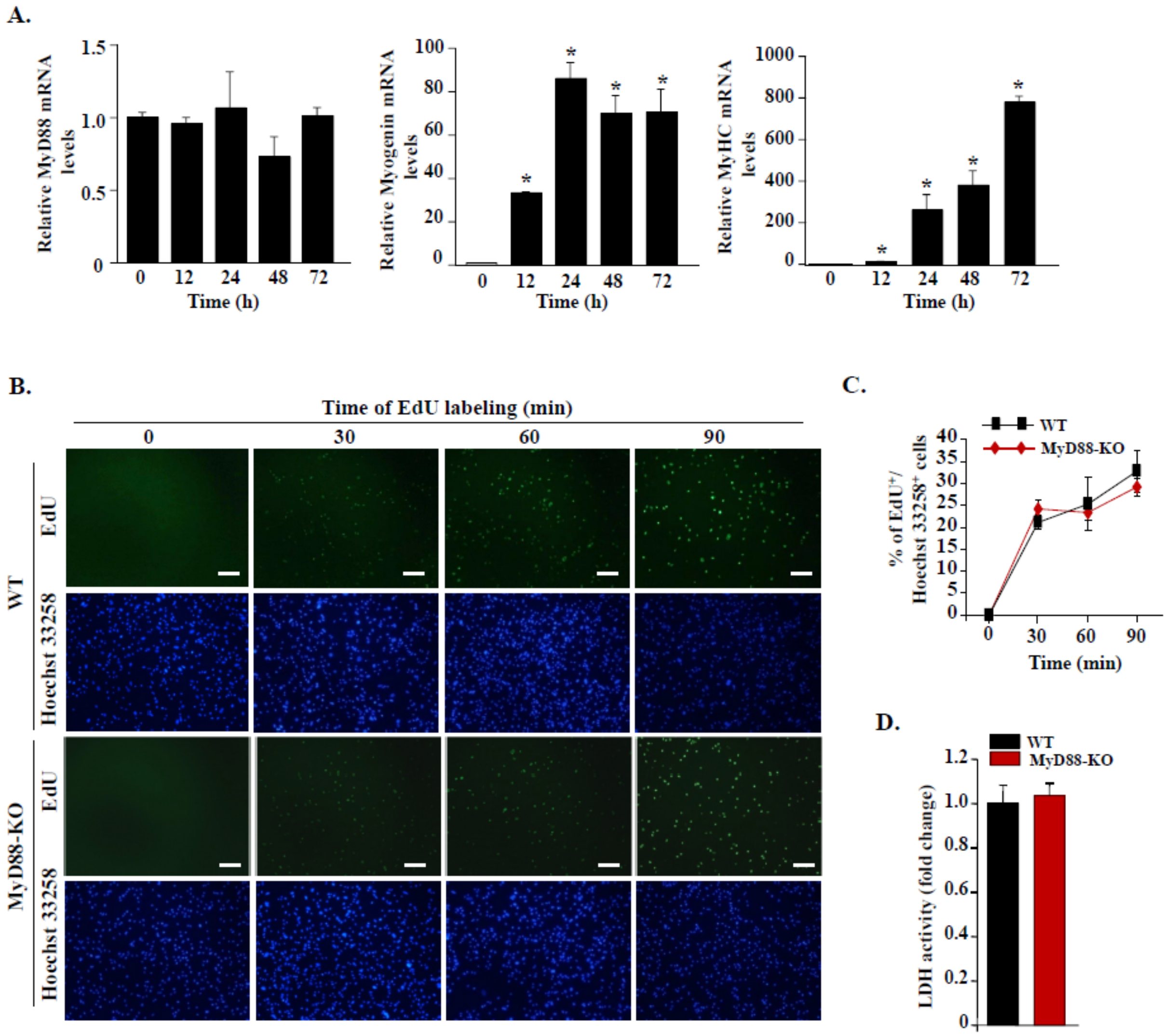

Supplementary Figure 5. Regulation of MyD88 and its role in the proliferation or survival of myogenic cells. (A) Primary WT myoblasts were incubated in DM for indicated time periods and the mRNA levels of MyD88 and differentiation markers, myogenin and MyHC, were measured by performing QRT-PCR. Data presented here show that there was no significant change in the mRNA levels of MyD88, whereas the levels of both myogenin and MyHC were significantly increased after incubation of myoblasts in DM. N=4 in each group. *p <0.05 from 0h by unpaired t-test. **(B)** WT and MyD88-KO primary myoblasts were plated at equal density and 24h later, cellular proliferation was measured using EdU incorporation assay for 30, 60 or 90

min. Nuclei were stained with Hoechst 33258 dye. Representative images of EdU^+ cells (green) and nuclei (blue) in WT and MyD88-KO cultures. Scale bar: 50 µm. **(C)** Quantification of percentage of EdU^+ cells in WT and MyD88-KO cultures. N=5 in each group. **(D)** WT and MyD88-KO primary myoblasts were incubated in DM for 48h and amounts of lactate dehydrogenase (LDH) in culture supernatants was measured. N=4 in each group. Error bars represent s.d.

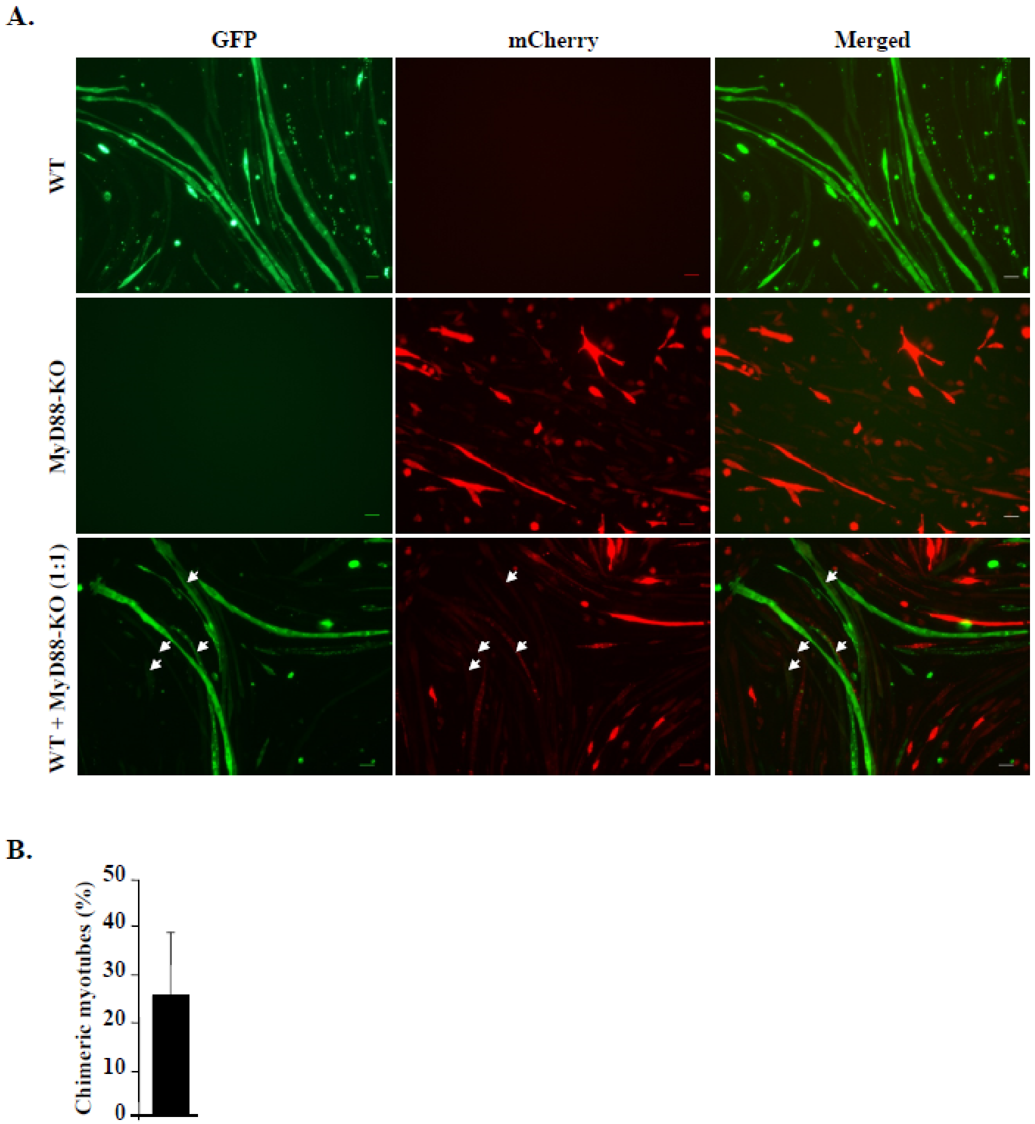

Supplementary Figure 6. Role of MyD88 in fusion partners. (A) WT myoblasts were transfected with a plasmid expressing GFP protein whereas MyD88-KO myoblasts were transfected with a plasmid expressing mCherry protein. After 36h of transfection, the cells were plated individually or mixed in 1:1 ratio at equal number and incubated in DM for an additional 48h. Representative images presented here demonstrate myotube formation in individual and mixed cultures. Scale bar: 20 µm. White arrows point to chimeric myotubes. **(B)** Quantification of chimeric myotubes in mixed cultures.

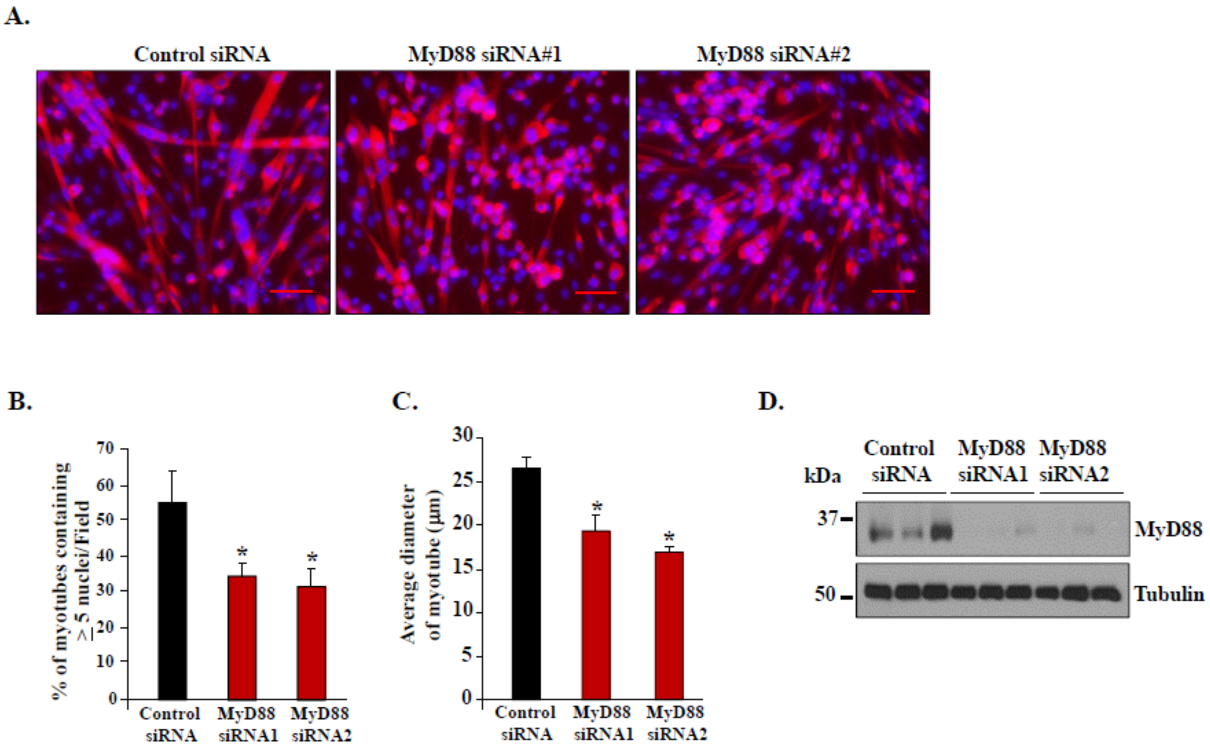

Supplementary Figure 7. RNAi-mediated knockdown of MyD88 inhibits myoblast fusion. **(A)** Primary WT myoblasts were transfected with scrambled (control) siRNA or two siRNA targeting different regions of MyD88 mRNA and incubated in DM for 48h. Representative photomicrographs of cultures after staining with anti-MyHC (red) and DAPI (blue). Scale bar: 50 μm. **(B)** Percentage of myotubes containing 5 or more nuclei at 48h after addition of DM. N=5 in each group. *$p<0.05$ from cultures transfected with control siRNA. **(C)** Average diameter of myotubes after 48h of addition of DM. N=5 in each group. Error bars represent s.d. *$p<0.05$ from cultures transfected with control siRNA. **(D)** Levels of MyD88 and unrelated protein tubulin in control and MyD88 siRNA-transfected cultures after 48h of incubation in DM.

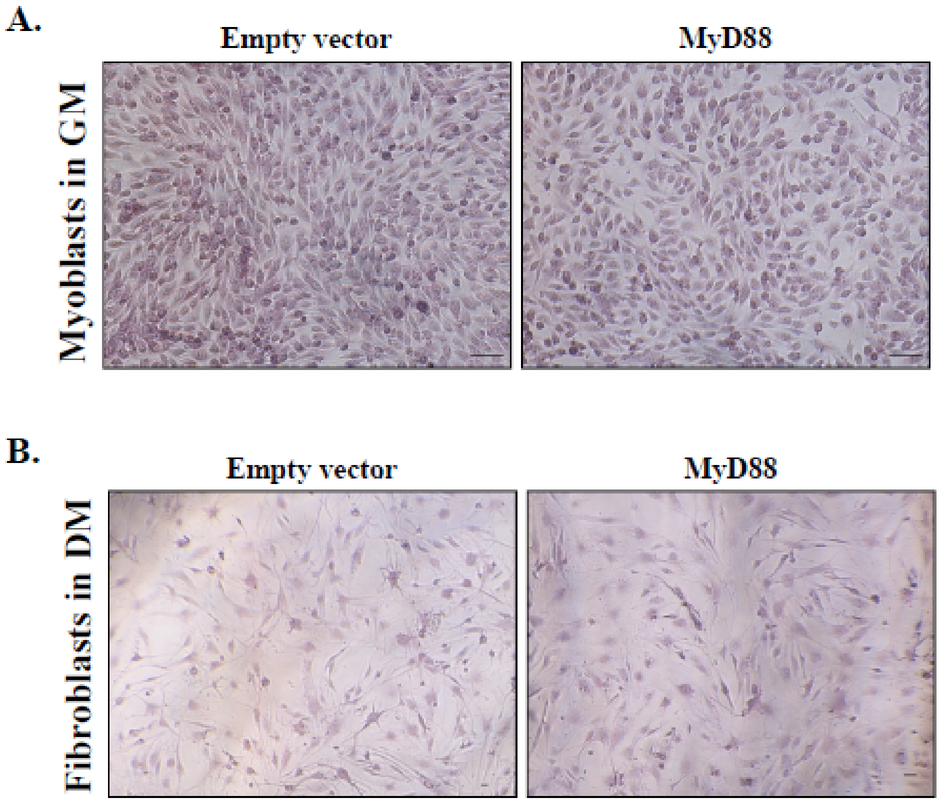

Supplementary Figure 8. MyD88 does not promote fusion in absence of a conventional myogenic differentiation program. Primary myoblast and fibroblast cultures were established from hind limb muscle of WT mice. **(A)** Myoblasts were transfected with empty vector or vector containing MyD88 cDNA and incubated in GM for 96h followed by staining with May-Grünwald solution. Representative images presented here demonstrate that overexpression of MyD88 has no effect on myoblast fusion in GM. Scale bar: 20 μm. **(B)** Primary mouse fibroblasts were transfected with empty vector or with MyD88 cDNA. The cells were then incubated in DM for an additional 72h followed by staining with May-Grünwald solution. Representative images presented here show that MyD88 overexpression did not cause fusion of fibroblasts in DM. Scale bar: 20 μm.

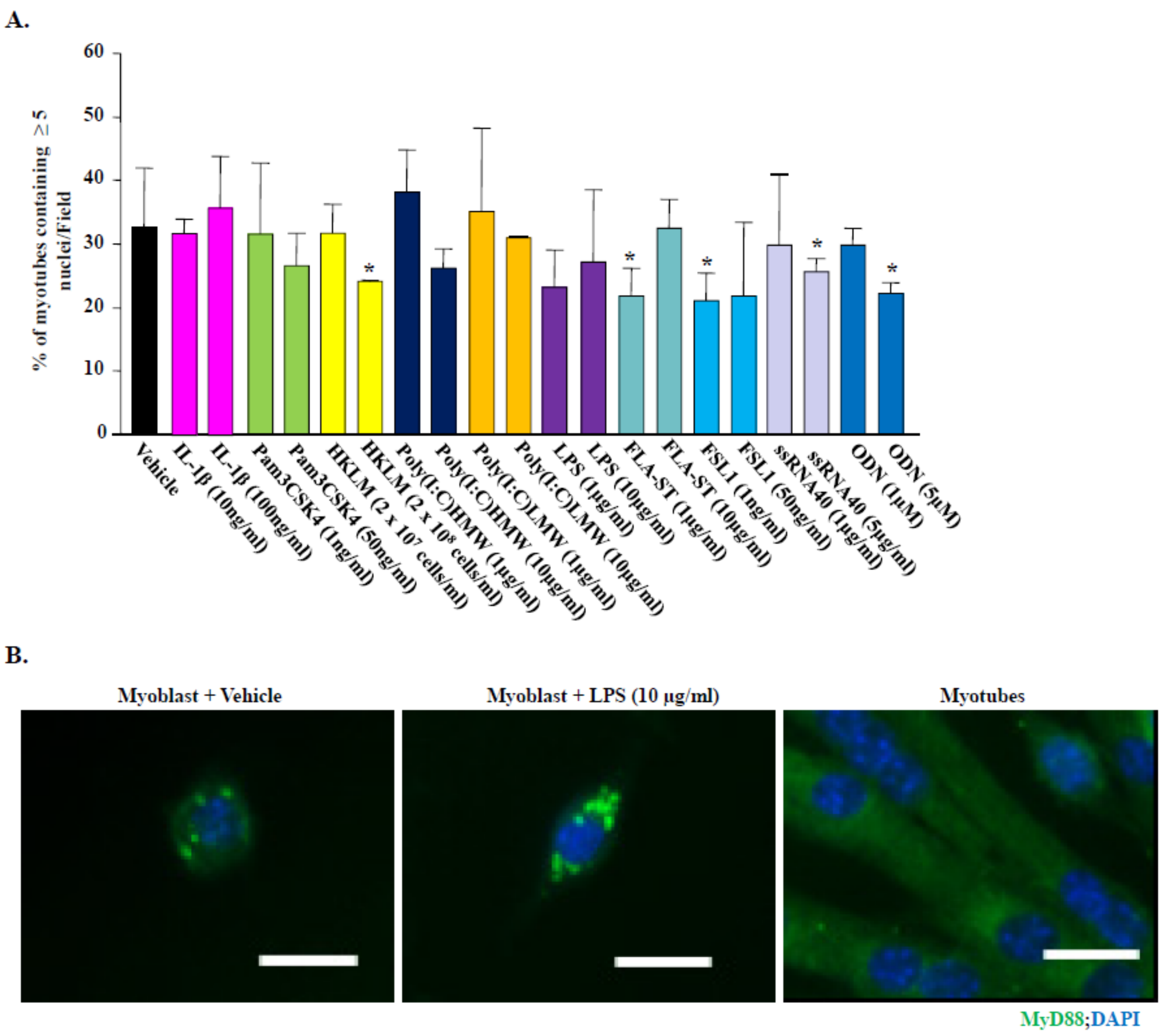

Supplementary Figure 9. IL-1β or agonist of TLRs do not stimulate myoblast fusion. (A) Primary WT myoblasts were incubated in DM with vehicle alone or indicated concentration of IL-1β, TLR1/2 agonist Pam3CSK4, TLR2 agonist HKLM, TLR3 agonists Poly(I:C) (high molecular weight) or Poly(I:C) (low molecular weight), TLR4 agonist lipopolysaccharide (LPS), TLR5 agonist FLA-ST (Flagellin from *S. typhimurium*), TLR6/2 agonist FSL1 (Pam2CGDPKHPKSF), TLR7/8 agonist ssRNA40, or TLR9 agonist ODN1826 for 48h. The cultures were stained for MyHC and nuclei were identified by staining with DAPI and percentage of myotubes containing 5 or more nuclei was recorded. N=4 in each group. Error bars represent s.d. *p<0.05 from cultures

treated with vehicle alone by unpaired t-test. **(B)** Primary WT myoblasts treated with vehicle alone or LPS or incubated in DM for 48h followed by staining with MyD88 antibody. Nuclei were counterstained with DAPI. Representative images presented here demonstrate that MyD88 is localized in the cytoplasm and forms aggregates in GM. The MyD88 aggregate formation is enhanced by treatment of myoblasts with LPS. Even though there is increased staining for MyD88, no aggregate formation was evident in myogenic cells incubated in DM for 48h. Scale bar: 20 μm.

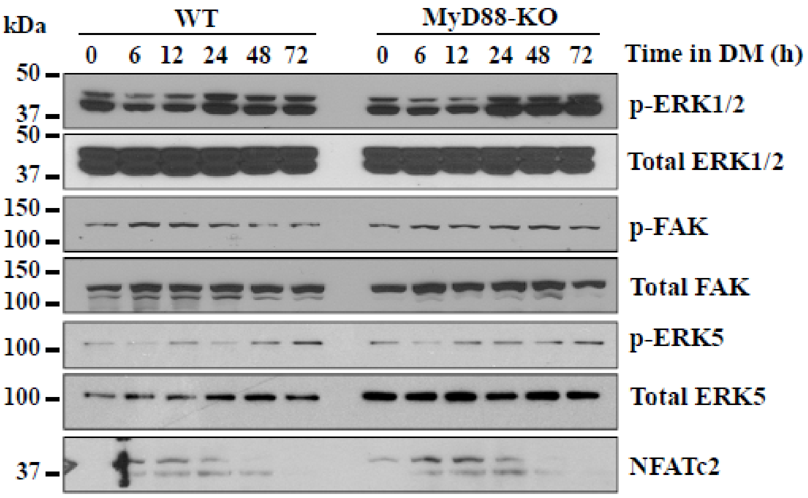

Supplementary Figure 10. Role of MyD88 in the activation of various profusion signaling pathways. Primary myoblasts prepared from hind limb muscle of WT and MyD88-KO mice were incubated in DM. Samples were collected at indicated time points following addition of DM and processed for biochemical analysis. Representative immunoblots demonstrate the levels of phospho-ERK1/2, total ERK1/2, phospho-FAK, total FAK, phospho-ERK5, total ERK5, and total NFATc2. This experiment was performed with same cell extracts as in the Fig. 4A in the main manuscript. Please refer to GAPDH blot in Fig. 4A as loading control.

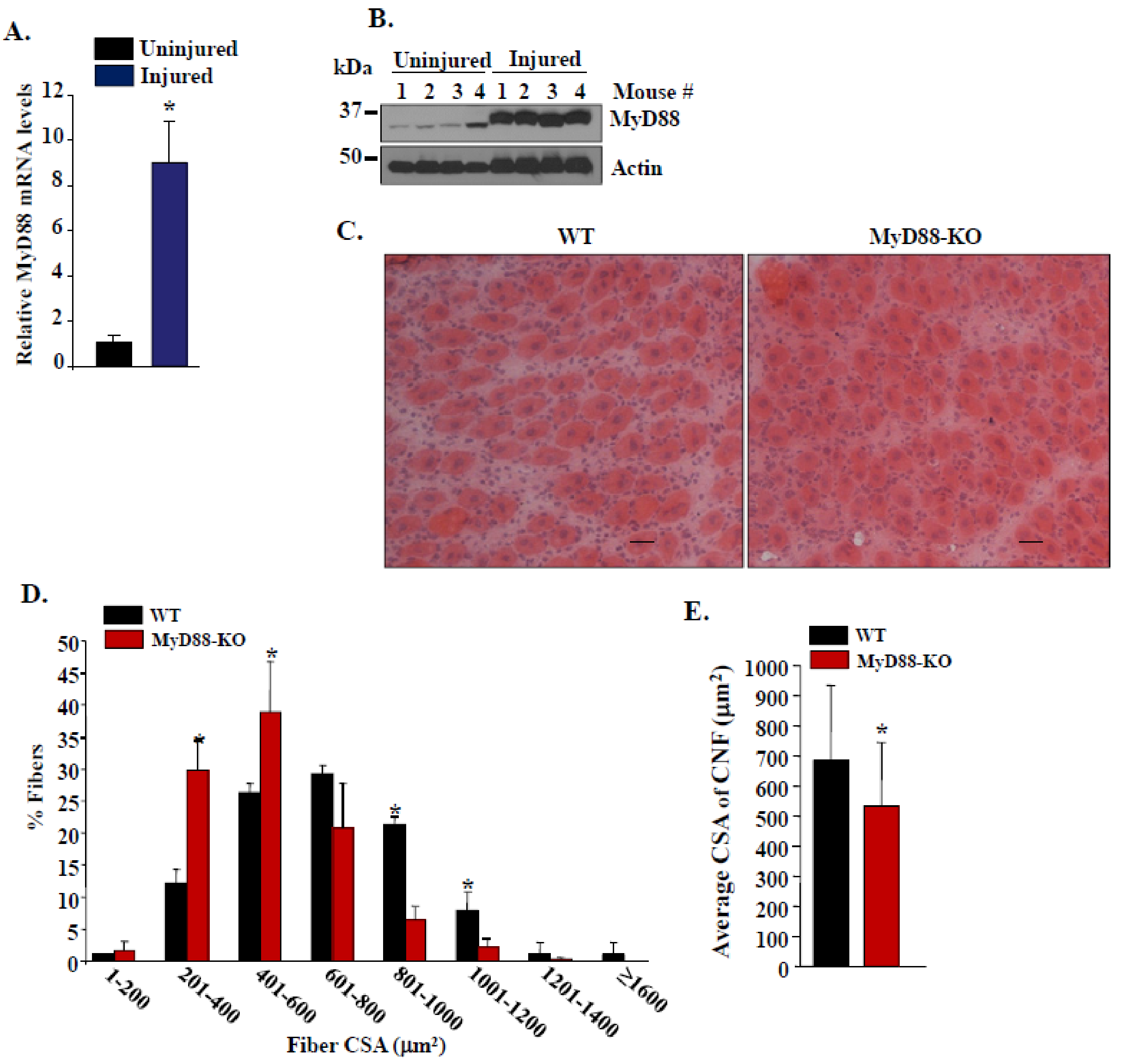

Supplementary Figure 11. Role of MyD88 in skeletal muscle regeneration. TA muscle of 12-week old WT mice was injected with 100 μl of 1.2% $BaCl_2$. After 3d, the muscles were collected and analyzed by QRT-PCR and western blot. **(A)** Relative mRNA levels of MyD88 in uninjured and injured TA muscle of WT mice are presented here. N=4 in each group. *p<0.01 from uninjured muscle. **(B)** Levels of MyD88 and unrelated protein actin in uninjured and injured TA muscle of mice. **(C)** TA muscle of WT and MyD88-KO mice was subjected to injury using 100 μl of 1.2% $BaCl_2$ solution. After 5d, the muscles were collected and processed for histological

analysis. Representative photomicrographs of H&E-stained TA muscle sections from 5d-injured WT and MyD88-KO mice. Scale bar: 50 µm. **(D)** Frequency distribution histogram representing myofiber CSA in TA muscle of WT and MyD88-KO mice at 5d after $BaCl_2$ injection. **(E)** Quantification of average myofiber CSA in 5d-injured TA muscle of WT and MyD88-KO mice. N=5 in each group. *$p<0.05$ from WT mice by unpaired t-test.

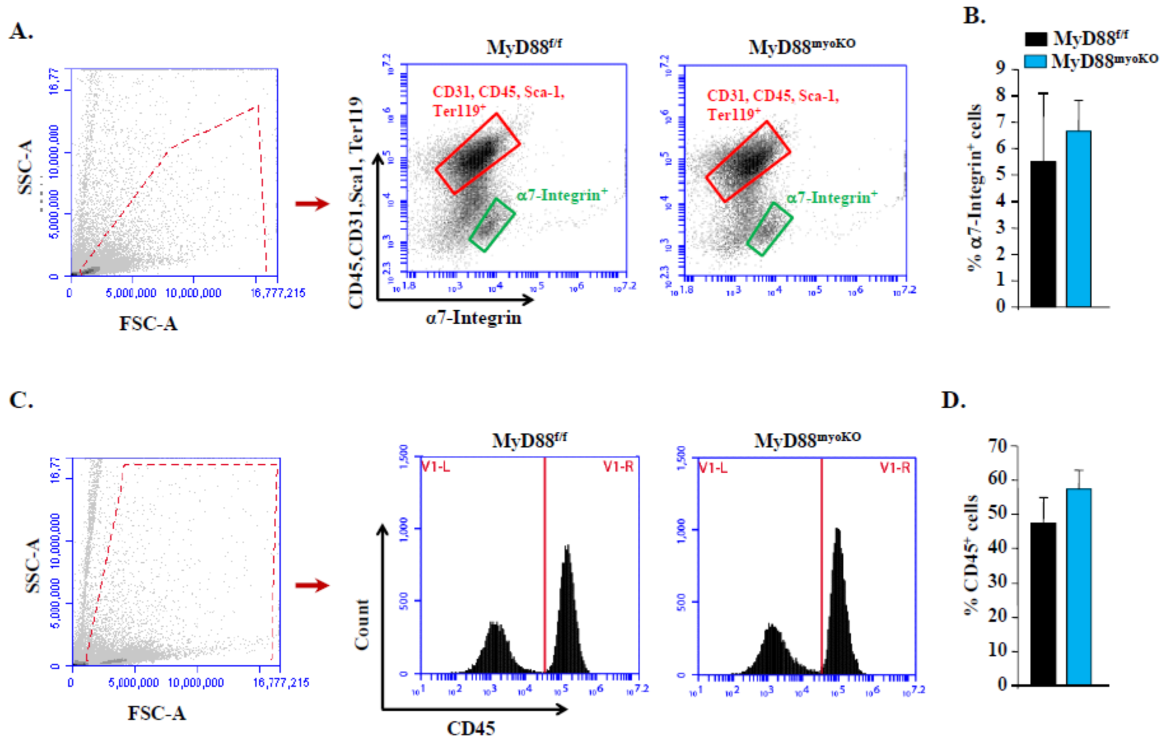

Supplementary Figure 12. Loss of MyD88 in myoblasts does not affect the number of satellite cells or inflammatory immune cells during regenerative myogenesis. TA muscle of 3-month old MyD88$^{f/f}$ and MyD88^{myoKO} mice was injured by intramuscular injection of 100 µl of 1.2% $BaCl_2$ solution. After 5d, the muscle was isolated and single cell suspension made was analyzed for satellite cells and inflammatory immune cells by FACS method using their specific cell surface markers. **(A)** Gating strategy and representative dot plots for satellite cells analyzed by FACS method. **(B)** Quantification of the percentage of α7-integrin^{+} satellite cells in 5d-injured TA muscle of MyD88$^{f/f}$ and MyD88^{myoKO} mice. **(C)** Gating strategy and representative histograms from FACS analysis showing proportion of CD45^{+} cells. **(D)** Quantification of the percentage of CD45^{+} cells in 5d-injured TA muscle of MyD88$^{f/f}$ and MyD88^{myoKO} mice. N=3 in each group. No statistical difference was observed between these two groups by unpaired t-test.

Fig. 1A.

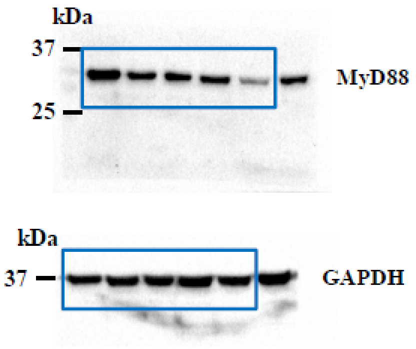

Fig. 1F.

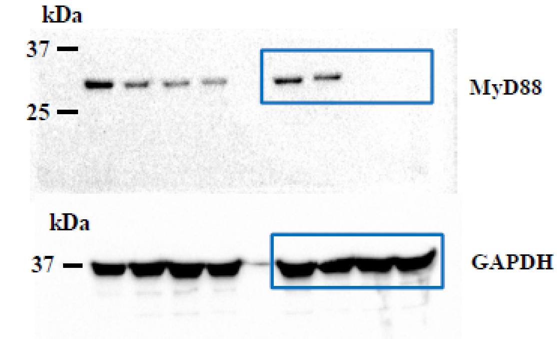

Fig. 1I.

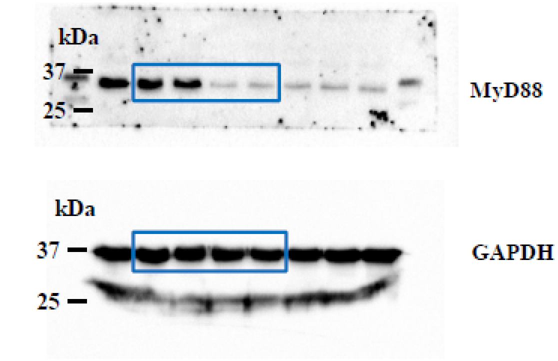

Fig. 2A.

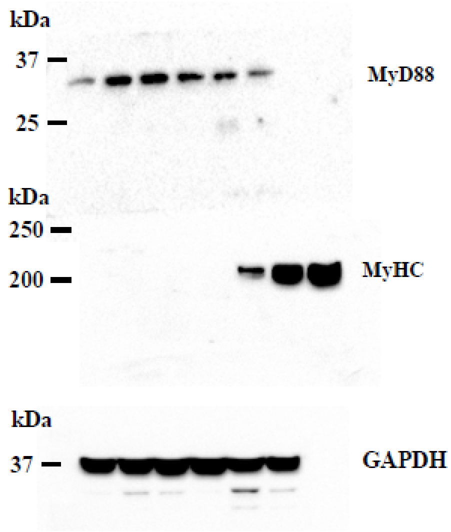

Fig 2H.

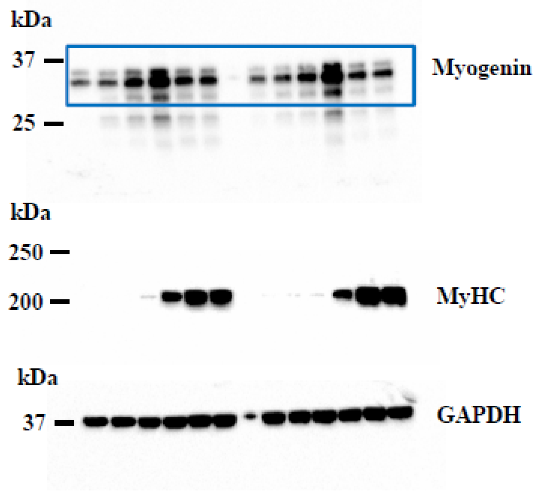

Fig. 3E.

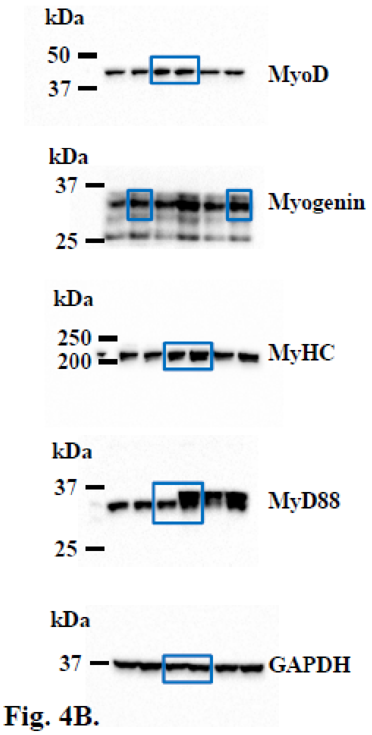

Fig. 4B.

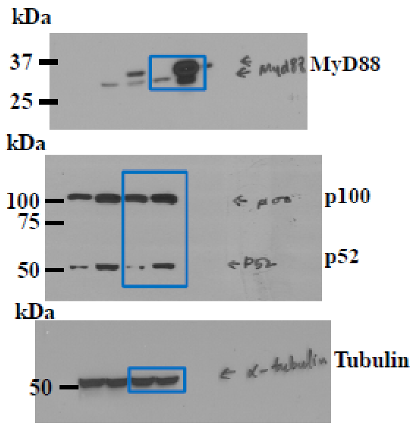

Fig. 4A.

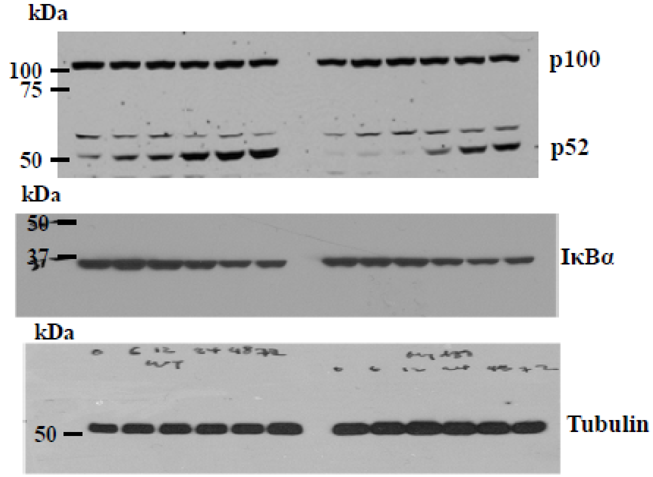

Fig. 4C.

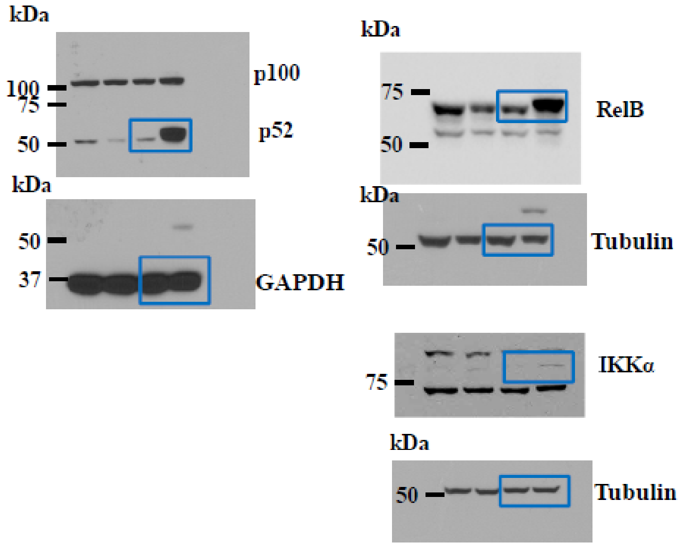

Fig. 5A.

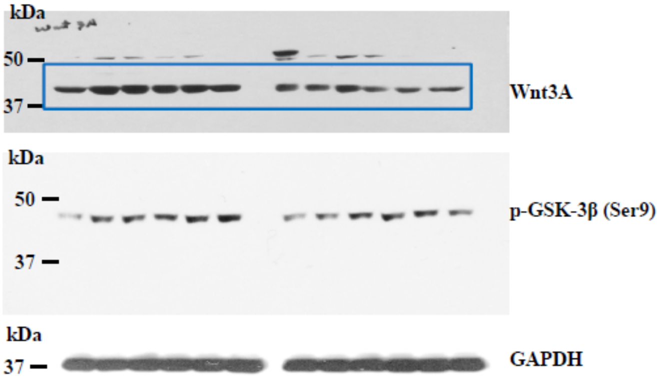

Fig. 5H.

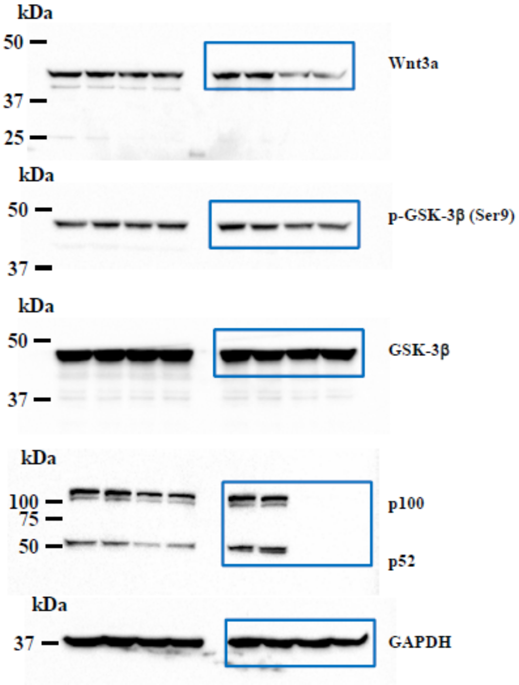

Fig. 8A.

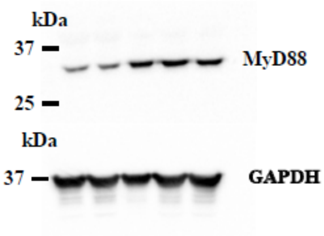

Fig. 5D (Supplementary)

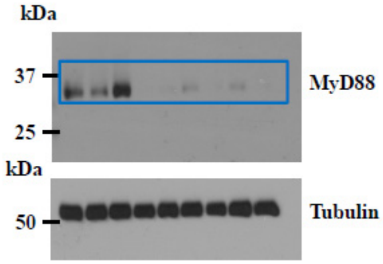

Fig. 9B (Supplementary)

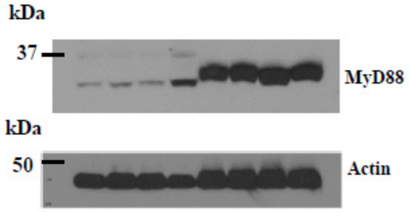

Fig. 8 (Supplementary)

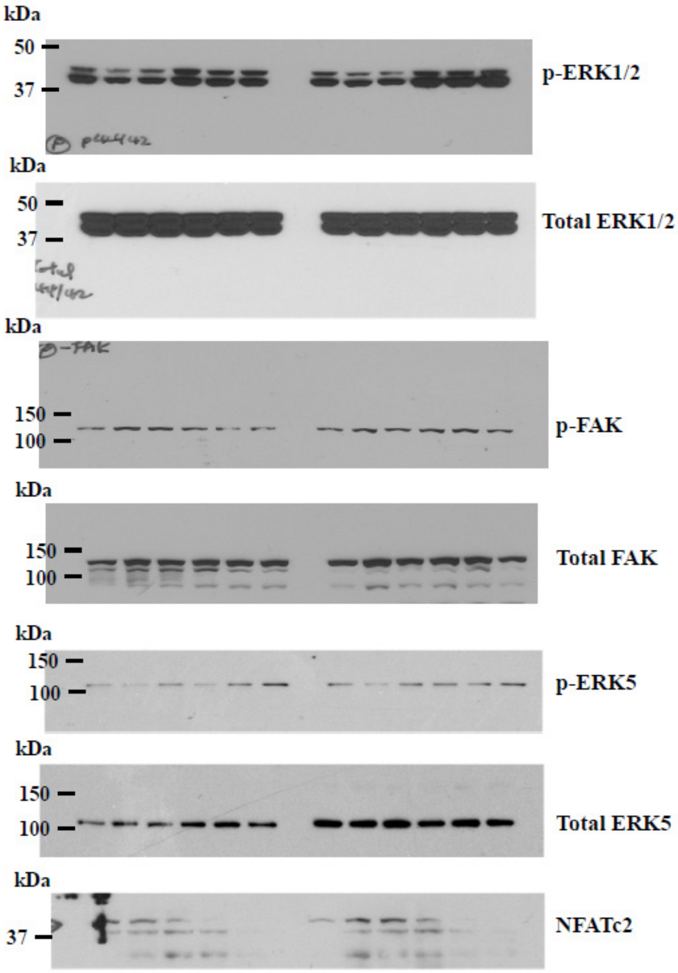

Supplementary Figure 13. Uncropped images of immunoblots with molecular weight markers. Original uncropped images of the immunoblots as displayed in the main and supplemental figures of the manuscript are presented here. Blue boxes were used to mark indicated bands where multiple bands appear in the same immunoblot. Molecular weight markers are presented on the left side of the immunoblots.

Supplementary Table 1: Antibodies and their dilution used in this study.

Antibody	Source	Dilution	Analysis
Polyclonal goat-anti-MyD88	R&D Systems # AF3109	1:500	WB
Monoclonal rabbit-anti-GAPDH	Cell Signaling Technology # 2118	1:2000	WB
Monoclonal mouse-anti-MyHC	DSHB MF20	1:1000	WB
Polyclonal rabbit-anti-Myogenin	Santa Cruz Biotechnology, sc-576	1:500	WB
Polyclonal rabbit-anti-MyoD	Santa Cruz Biotechnology, sc-304	1:500	WB
Polyclonal rabbit-anti-p100/p52	Cell Signaling Technology # 4882	1:500	WB
Monoclonal rabbit-anti-phospho-IκBα	Cell Signaling Technology # 2859	1:500	WB
Monoclonal rabbit-anti-total IκBα	Cell Signaling Technology # 4812	1:500	WB
Monoclonal rabbit anti-α-Tubulin	Cell Signaling Technology # 2125	1:2000	WB
Monoclonal rabbit anti-RelB	Cell Signaling Technology # 10544	1:500	WB
Polyclonal rabbit anti-total IKKα	Cell Signaling Technology # 2682	1:500	WB
Polyclonal rabbit anti-Wnt3a	Millipore # ABD124	1:500	WB
Monoclonal rabbit anti-phospho-GSK-3β	Cell Signaling Technology # 5558	1:500	WB
Monoclonal rabbit-anti-total GSK-3β	Cell Signaling Technology # 2859	1:500	WB
Polyclonal rabbit-anti-phospho-ERK1/2	Cell Signaling Technology # 9101	1:500	WB
Polyclonal rabbit-anti-total ERK1/2	Cell Signaling Technology # 9102	1:500	WB
Polyclonal rabbit-anti-phospho-FAK	Cell Signaling Technology # 3281	1:500	WB
Monoclonal rabbit-anti-total FAK	Cell Signaling Technology # 13009	1:500	WB
Polyclonal rabbit-anti-phospho-ERK5	Cell Signaling Technology # 3371	1:500	WB
Polyclonal rabbit-anti-total ERK5	Cell Signaling Technology # 3372	1:500	WB
Monoclonal mouse anti-NFATc2	Novus Biologicals # NB300-504	1:500	WB
Polyclonal rabbit-anti-Dystrophin	Abcam # b15277	1:400	Immunohistochemistry
Monoclonal mouse-anti-MyHC	DSHB MF20	1:200	Immunocytochemistry
Monoclonal mouse-anti-eMyHC	DSHB F1.652	1:10	Immunohistochemistry
Polyclonal rabbit-anti-Laminin	Sigma #L9393	1:500	Immunohistochemistry
Monoclonal mouse-anti-Pax7	DSHB Pax7	1:10	Immunohistochemistry
Polyclonal goat-anti-MyD88	R&D Systems # AF3109	1:80	Immunocytochemistry
Monoclonal mouse-anti-Integrin α7	Miltenyi Biotec # 130-102-717	1:10	FACS
Monoclonal mouse-anti-CD31	eBioscience # 12-0311-82	1:428	FACS
Monoclonal mouse-anti-CD45	eBioscience # 12-0451-83	1:428	FACS
Monoclonal mouse-anti-Sca1	eBioscience # 12-5981-82	1:428	FACS
Monoclonal mouse-anti-Ter-119	eBioscience # 12-5921-82	1:428	FACS

Supplementary Table 2. Sequence of the primers used for QRT-PCR analyses.

Gene	Sequence (5' → 3')
Myogenin	CATCCAGTACATTGAGCGCCTA (Forward)<br>GAGCAAATGATCTCCTGGGTTG (Reverse)
Myh4	CGGCAATGAGTACGTCACCAAA (Forward)<br>TCAAAGCCAGCGATGTCCAA (Reverse)
Myd88	TATCGCTGTTCTTGAACCCTCG (Forward)<br>AGGCATCCAACAAACTGCGA (Reverse)
β1-Integrin	CATCCCAATTGTAGCAGGCG (Forward)<br>CGTGTCCCACTTGGCATTCAT (Reverse)
β1D-Integrin	CATCCCAATTGTAGCAGGCG (Forward)<br>GAGACCAGCTTTACGTCCATAG (Reverse)
Adam12	GGGCAAGAAGGCATAAGAGAGAGA (Forward)<br>TGGTGAATGGGTCCTGGCTTAT (Reverse)
Myomaker	TATACTCCGGTCCCATAGGC (Forward)<br>ATGCTCTTGTCGGGGTACAG (Reverse)
M-cadherin	TGGGCAGTCCCTGAGCCCAAA (Forward)<br>TCCAGCGTGGCATTGAGGTACA (Reverse)
N-cadherin	CAGCAGATTTCAAGGTGGACGA (Forward)<br>TCCTGGGTTTCTTTGTCTTGGG (Reverse)
Myoferlin	CTACCAGAATGAGAATCGCTACCC (Forward)<br>TACTCCCAGCCTTTCTCATCCA (Reverse)
Nephronectin	CCAGAACAACTCCACTACCACCAA (Forward)<br>CTGGGTCGTCCTTTACTTCCTCAT (Reverse)
MOR23	GCTGGTGGTCGTGGCTGTATTTAT (Forward)<br>AACCCAAGGCAATGCAGAAGTG (Reverse)
Caveolin-3	GACCCCAAGAACATCAATGAGGAC (Forward)<br>AGAAGGAGATACAGGCGAACAGGA (Reverse)
IL-4	GGATGTGCCAAACGTCCTC (Forward)<br>GAGTTCTTCTTCAAGCATGGAG (Reverse)
IL-6	CCTTCTTGGGACTGATGCTGG (Forward)<br>GCCTCCGACTTGTGAAGTGGT (Reverse)
β-actin	CAGGCATTGCTGACAGGATG (Forward)<br>TGCTGATCCACATCTGCTGG (Reverse)
